# *In vivo* evaluation and material basis of the bi-directional immune modulatory effect of *Scutellaria barbata* D. Don extract

**DOI:** 10.3389/fmed.2026.1797041

**Published:** 2026-04-07

**Authors:** Kerui Wu, Yan Ran, Zinuo Chen, Chenglong Chen, Xuran Gu, Qinghua Cui, Ruikun Du

**Affiliations:** Qingdao Academy of Chinese Medical Sciences, Shandong University of Traditional Chinese Medicine, Qingdao, China

**Keywords:** antiviral innate immunity, bi-directional immune modulation, broad-spectrum antiviral, Chinese herbal medicine, *Scutellaria barbata* D. Don

## Abstract

The innate immunity of host defends universally against various invading viruses, providing attractive targets for development of novel broad-spectrum antivirals. However, the antiviral innate immunity usually has “double-edged sword” effects, and clinical applications of either innate immune enhancers or suppressors depend highly on the timing of administration, which requires precision diagnosis and careful evaluation of the disease status. Irrational use of a single-directional immune modulator may greatly compromise the efficacy or even cause more severe diseases. The bi-directional modulators can balance the protective-vs.-detrimental effects of antiviral innate immunities, showing expanded “window of therapeutic opportunity” and advanced translational potency. Previously, we identified the potential bi-directional immune modulatory effect of *Scutellaria barbata* D. Don (SBD) *in vitro*. In the present study, we further validated that SBD maintained a relatively stable proportion of proinflammatory polarization among the lung macrophages of mice, regardless of with or without LPS stimulation, in a mode of bi-directional modulation. In addition, we explored the material basis underlying the bi-directional immune modulatory effect of SBD. Our study consolidated the bi-directional immune modulatory property of SBD and shed light on the development of next-generation innate immune modulatory agents.

## 1 Introduction

Virus infections have caused a substantial burden on public health globally, while our antiviral countermeasures remain limited (1). Due to the high diversity of pathogenic viruses, the direct-acting antiviral (DAA) agents were usually developed based on a “one-bug, one drug” paradigm, harboring effectiveness against a single viral genotype only or a few closely related viruses at best (2). While the host-targeting antiviral agents, especially those antiviral immune modulators, may possess potential broad-spectrum antiviral (BSA) property of being effective against a wide range of viruses, offering promising therapeutic solutions for the largely unmet need in treating both existing and emerging viral infections (2).

However, the antiviral immunity usually plays as a “double-edged sword” during virus infections (3). On one hand, a timely and effective immune response is critical for the host to restrict and/or eliminate invading viruses extensively, and it is straightforward to assume

that an immune enhancer can improve the antiviral property of hosts. One the other hand, excessive immune responses may result in uncontrolled inflammation and tissue damage, in these situations, administration of immune suppressors instead may provide beneficial effects (3). It is therefore critical to note that the effectiveness of these single-directional immune modulators depends highly on the timing of administration, which requires precision diagnosis and careful evaluation of the disease status, while irrational applications may greatly compromise the drug efficacy or even cause more severe diseases (4, 5).

Previously, we reported the potential bi-directional immune modulatory effect of *Scutellaria barbata* D. Don (SBD) using a lipopolysaccharide (LPS) induced inflammation model based on the mouse macrophage Raw264.7 cells (6). In the absence of LPS stimulation, SBD treatment can directly promote the Raw264.7 to establish a proinflammatory status. While in the presence of increasing concentrations of LPS, as the LPS-induced cellular inflammation response increase, the proinflammatory effect of SBD became attenuated gradually and ultimately converted to a climbing anti-inflammatory property, facilitating the Raw264.7 cells to maintain a relatively stable proinflammatory status regardless of the extent of external stimulus (6). It can be speculated that SBD may have a much wider “window of therapeutic opportunity” compared to single-directional immune enhancers or suppressors, exhibiting more advanced value and translational potency.

In the present study, we further examined the bi-directional immune modulatory effect of SBD *in vivo*, and unraveled the underlying material basis.

## 2 Method

### 2.1 Ethics statement

All animal procedures were performed in accordance with protocols approved by the Institutional Animal Care and Use Committee (IACUC) of Shandong University of Traditional Chinese Medicine (Approval: SDUTCM20250920425).

### 2.2 Cells and reagents

The mouse monocyte macrophage leukemia cells (Raw264.7) were cultured in Dulbecco's modified Eagle medium (DMEM) supplemented with 10% fetal bovine serum (FBS) and 1% penicillin/streptomycin (10,000 U/ml). The compounds moslosooflavone, dinatin, wogonin, quercetin, baicalin, luteolin and salvigenin were purchased from MedChemExpress (MCE). The extract of SBD was prepared and stored as previously described.

### 2.3 Animal models of acute lung injury (ALI)

Mouse model of ALI was established as described previously (7). In brief, female BALB/c mice (4–6 weeks old) were intranasally administered with 50 μg of *Escherichia coli* O55:B55 LPS (Sigma) in 25 μl PBS induce ALI. After 6 h stimulation, the mice were sacrificed and the lung tissues were exercised for further analysis. The right lower lung lobe of each mouse was fixed for histopathologic analysis. The left lung tissues were homogenized, with a proportion subjected to qRT-PCR and the others for flow cytometry analysis. The primers used for qPCR analysis are listed in Supplementary Table 1.

### 2.4 Hematoxylin-and-eosin (H&E) staining

The mouse lungs were dissected and fixed in 4% formaldehyde and embedded in paraffin. The embedded tissues were cut into 5 μm thick sections and stained with H&E. The stained sections were viewed and captured using a BX53 microscope (Olympus, Japan).

### 2.5 Flow cytometry

Total single cell suspensions prepared from the mice lungs were stained using fluorochrome-conjugated antibodies against molecules CD45, CD11b, F4/80, and CD86. Viability dye (eBioscience, USA) was deployed to eliminate dead cells. Flow cytometry was conducted by using an LSR II D324 system (BD Biosciences, USA). Live cells were gated to analyze macrophages (CD45^+^CD11^+^F4/80^+^) and their proinflammatory polarization (CD86^+^) using FlowJo (Tree Star, USA).

### 2.6 Network pharmacology-guided identification of potential active components of SBD

According to the Chinese medicine system pharmacological database and analysis platform (TCMSP, https://ngdc.cncb.ac.cn/databasecommons/database/id/4096), 94 compounds have been recorded as SBD constituents, of which 29 have oral bioavailability (OB) ≥ 30% and druglikeness (DL) ≥ 0.18 (8). The potential targets of these 29 active components were acquired using Swiss Target Prediction (http://www.swisstargetprediction.ch), and the network between SBD active ingredients and the potential targets were drawn using Cytoscape3.9. The parameters including degree, betweenness centrality (BC), closeness centrality (CC) and average shortest path length (ASPL) of each active component were calculated. The top 10 components representing the most concentrated nodes in the network were identified as the most potential active components of SBD.

### 2.7 RAW264.7 cell-based inflammation model

To examine the potential regulatory effect of active components derived from SBD on inflammation, the RAW264.7 cells were left untreated or treated with LPS (10 ng/mL), in presence of increasing concentrations of indicated compounds. After 8 h-incubation, the cells were harvested to determine the expression level of a panel of pro-inflammatory cytokines using qRT-PCR analysis as described previously.

## 3 Results

### 3.1 SBD exhibits bi-directional modulatory effect on inflammation response *in vivo*

In order to examine the potential bi-directional immune modulatory effect of SBD, the BALB/c mice were separately administered with PBS or SBD (4 g/kg/day) for 2 days, then left untreated or stimulated with LPS to induce ALI. Besides, mice administered with dexamethasone (DXM) as a positive control, was administered at 5 mg/kg and 2 h before LPS stimulation. After 6 h of stimulation, the mice were sacrificed and the lung tissues were excised.

**Figure 1:**
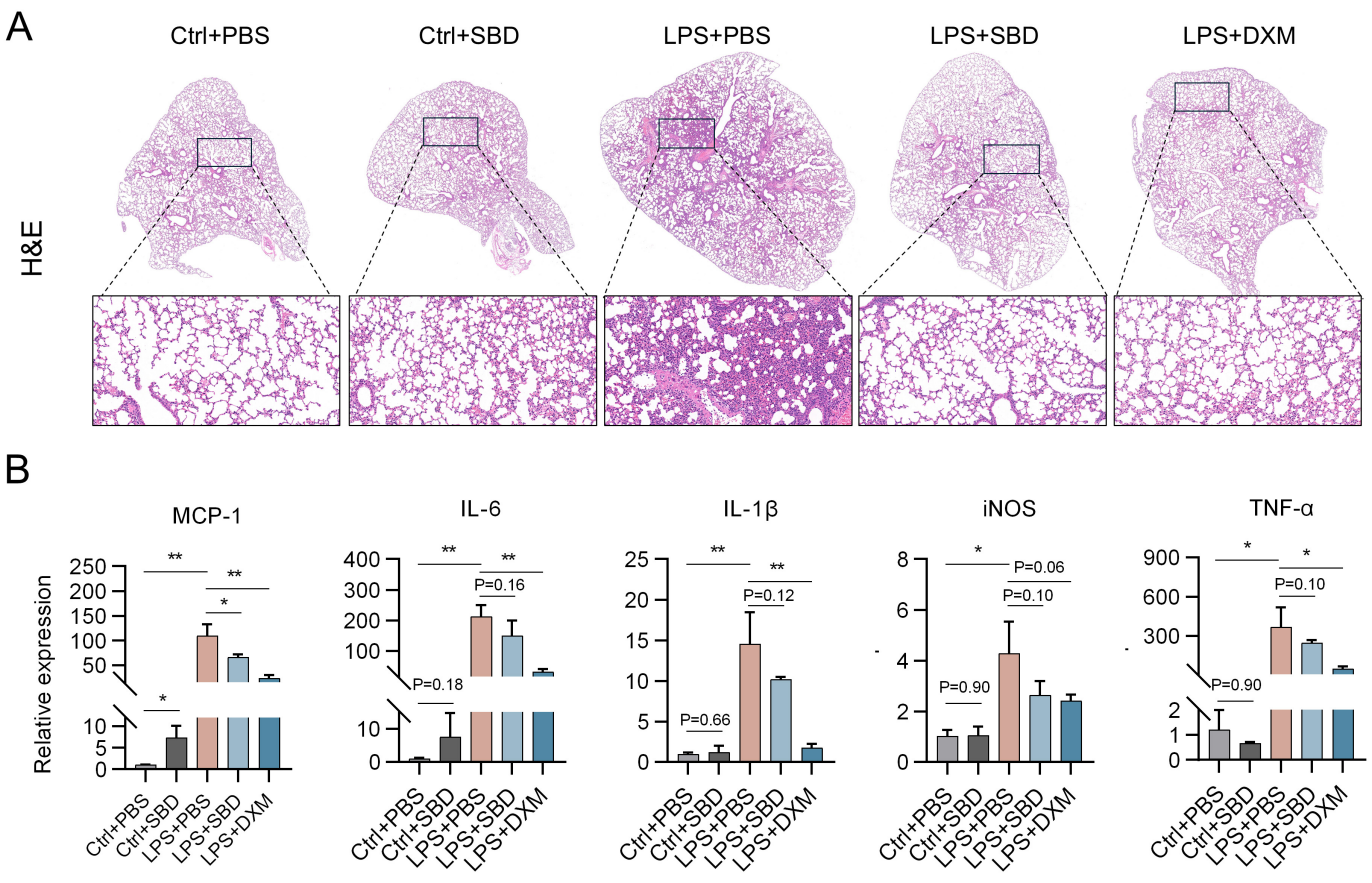
Bi-directional regulation of *Scutellaria barbata* D. Don (SBD) on the inflammation response in mice lungs. BALB/c mice were mock or intranasally challenged with LPS, establishing models of control or acute lung injury (ALI), respectively. For SBD treatment, both control or ALI mice were administered with SBD (4 g/kg/day) at intervals of twice daily and starting at 2 days prior to LPS stimulation. Mice received PBS in parallel was used as negative control. While ALI mice receiving dexamethasone (DXM) at 2 h before LPS stimulation were used as positive controls. At 6 h after LPS challenge, the mice were sacrificed and the lungs were excised for further analysis. **(A)** Histopathological changes of mice lungs. **(B)** Relative expression of proinflammatory intermediators in mice lungs. Data were expressed as mean ± standard deviation (SD) of 3 replicates and analyzed by unpaired students' *t* test. *, *p* < 0.05; **, *p* < 0.01.

The right lower lobe of each mouse lung was fixed for histopathological analysis using H&E staining (Figure 1A). As expected, it was observed that SBD can directly induce a mild pro-inflammation status in healthy mice lungs, displaying slight thickening of the alveolar wall and some extent of inflammatory cell infiltration. Conversely, since the lungs from the LPS-stimulated mice displayed severe histopathological changes, SBD treatment markedly alleviated the ALI, of which the efficacy is comparable to that of DXM.

Another portion of the mice lungs were removed to examine the expression level of various proinflammatory mediators using qRT-PCR, including monocyte chemoattractant protein-1 (MCP-1), interleukin (IL)-6, IL-1β, inducible nitric oxide synthase (iNOS), and tumor necrosis factor (TNF)-α. As shown in Figure 1B, the expression pattern of MCP-1 exhibited a typical bi-directional regulation by SBD, that SBD administration significantly increased the expression of indicated gene in healthy mice lungs, while in LPS-induced ALI models, SBD remarkably suppressed the LPS induced MCP-1 expression. While for the other proinflammatory mediators, the expression patterns upon LPS and/or SBD treatment showed similar tendency to that of MCP-1, although with weak statistical significances (Figure 1B).

Taken together, our data clearly demonstrated the bi-directional modulatory effect of SBD on host inflammation *in vivo*.

### 3.2 SBD exhibits bi-directional modulatory effect on inflammatory polarization of macrophages

**Figure 2:**
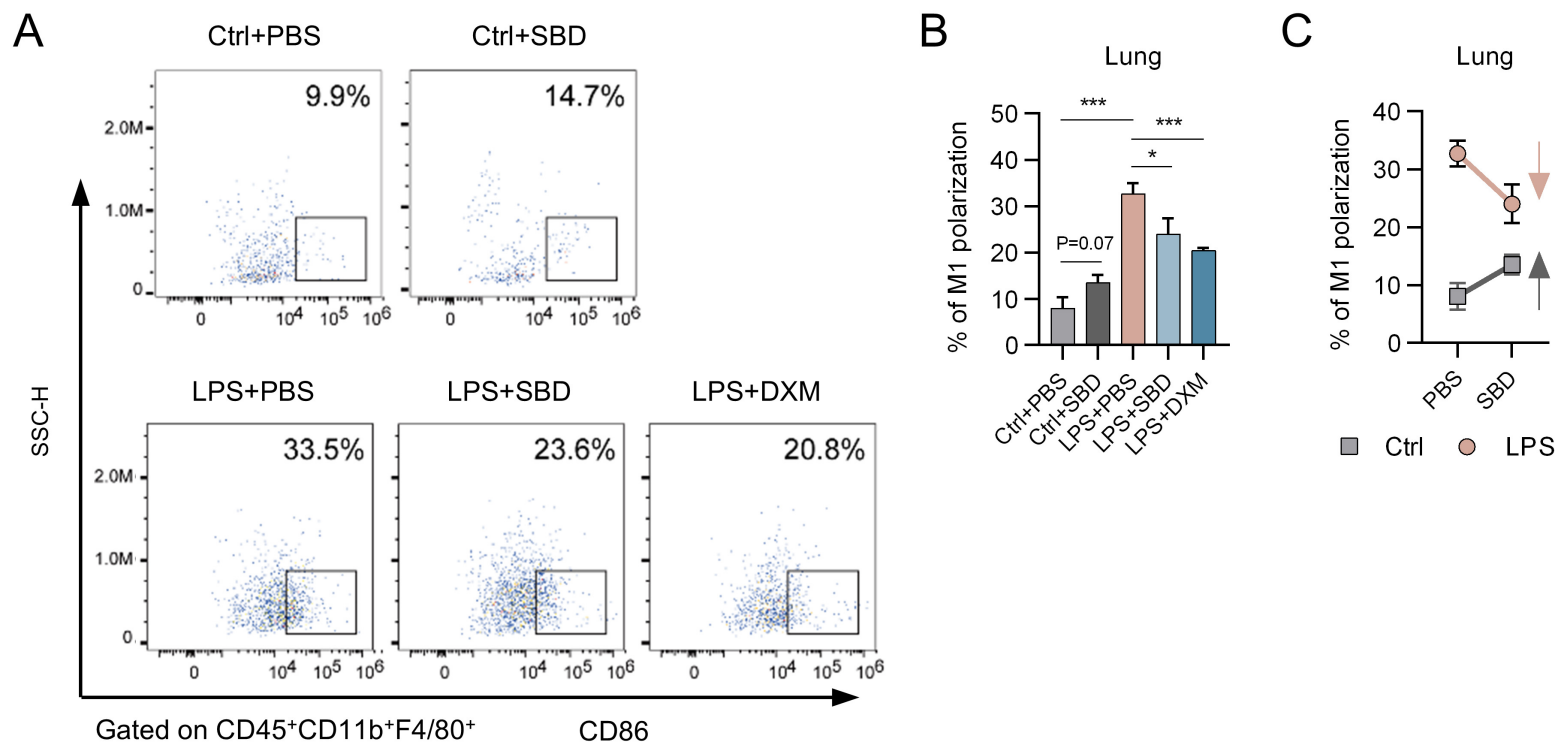
Bi-directional regulation of *Scutellaria barbata* D. Don (SBD) on proinflammatory polarization of lung macrophages. The control or LPS-induced ALI mice, in the absence or presence of SBD treatment, were sacrificed at 6 h after LPS stimulation and the lungs were subjected to flow cytometry analysis. ALI mice receiving dexamethasone (DXM) at 2 h before LPS stimulation were used as positive controls. **(A)** Flow cytometry analysis of the proinflammatory phenotypes in total lung macrophages. **(B)** The proportion of inflammatory macrophages in mice lungs. **(C)** The regulatory effect of SBD on the polarization of inflammatory macrophage in lungs from healthy and ALI mice. Data were expressed as mean ± standard deviation (SD) of 3 replicates and analyzed by unpaired students' *t* test. *, *p* < 0.05; ***, *p* < 0.001.

The proinflammatory macrophages (also known as M1) contribute predominately to the production of proinflammatory cytokines in the LPS-induced ALI mice (9). We next sought to determine the effect of SBD on the M1 polarization in mice lungs with or without LPS stimulation. The total macrophages in mice lungs were gated on CD45^+^CD11b^+^F4/80^+^ and the CD86^+^ were recognized as M1 polarized cells (Figure 2A and Supplementary Figure 1). As shown in Figure 2B, SBD treatment obviously elevated (*p* = 0.07) the proportion of M1 phenotypes among total macrophages in healthy mice lungs. On the contrary, as LPS induced a much more remarkable increase in M1 polarization, SBD greatly attenuated this phenotypic transition (*p* < 0.05). These results suggested that SBD exerts its bi-directional immune modulatory probably by balancing the proportion of inflammatory polarized macrophages (Figure 2C).

### 3.3 SBD exhibits bi-directional immune modulatory effect by multi-components and multi-modes of action

The Chinese herbal medicines have been well characterized by multi-components and multi-targets (10). In order to better elucidate the material basis underlying the bi-directional immune modulatory property of SBD, the 94 active components of SBD recorded in the TCMSP were screened based on the characteristics of OB ≥ 30% and DL ≥ 0.18. As a result, 29 constituents were selected and subjected to Swiss Target Prediction to identify potential targets. The active ingredient-target network diagram of SBD was drawn using Cytoscape3.9, generating 358 nodes and 2068 edges (Supplementary Figure 2). Table 1 listed the top 10 components representing the most concentrated nodes in the network, of which 7 are commercially available and were purchased for further analysis. The chemical structures of these 7 components were shown in Supplementary Figure 3.

**Table 1:** Top 10 most active components of SBD predicted by network-pharmacology analysis.

Compound name	Degree	BC	CC	ASPL
Dinatin^*^	23	0.04	0.46	2.16
5-hydroxy-7,8-dimethoxy-2-(4-methoxyphenyl)chromone	23	0.06	0.46	2.16
Moslosooflavone^*^	22	0.06	0.46	2.18
Luteolin^*^	22	0.03	0.46	2.18
Baicalein^*^	22	0.05	0.46	2.18
Salvigenin^*^	21	0.03	0.45	2.20
Chrysin-5-methylether	21	0.03	0.45	2.20
Wogonin^*^	20	0.07	0.45	2.23
7-hydroxy-5,8-dimethoxy-2-phenyl-chromone	20	0.03	0.45	2.23
Quercetin^*^	19	0.02	0.44	2.25

^*^, means commercially available. BC, betweenness centrality; CC, closeness centrality; ASPL, average shortest path length.

**Figure 3:**
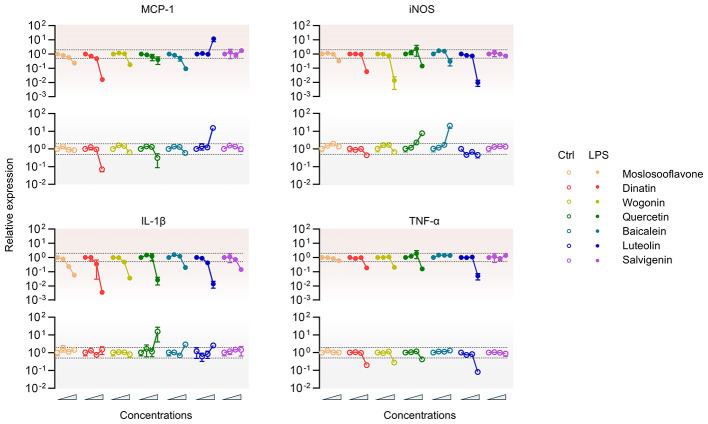
The regulatory effect of potential active components of SBD on inflammation. Raw264.7 cells were treated with increasing concentrations of indicated compounds in the absence or presence of LPS stimulation. After 8 h incubation, the cells were harvested and the relative expression of various proinflammatory mediators were examined using qRT-PCR analysis.

Next, the potential regulatory effect of these 7 active components of SBD on the inflammation response was examined using the Raw264.7 cell-based inflammation model (6). To this purpose, Raw264.7 cells were treated with increasing concentrations of test compounds in the absence or presence of LPS stimulation. After 8 h incubation, the cells were harvested and the relative expression of proinflammatory mediators, including MCP-1, iNOS, IL-1β, and TNF-α were examined using qRT-PCR analysis. As a result, all seven components showed reasonable impacts on the inflammatory response, albeit in different patterns (Figure 3).

(1) Moslosooflavone, dinatin, wogonin remarkably suppressed the LPS-induced expression of all the four tested proinflammatory mediators. While in the absence of LPS, the three compounds showed either little effect or downregulation on the statuses of indicated proinflammatory factors.
(2) Quercetin and baicalin exhibited obvious bi-directional modulatory mode of action on the expression of iNOS and IL-1β, but not for MCP-1 and TNF-α. Instead, quercetin tended to inhibit the expression of both MCP-1 and TNF-α either in the absence or presence of LPS stimulation, while baicalin showed inhibitory effect on LPS induced but not baseline MCP-1 expression. Note that baicalin exhibited no effect on the expression of TNF-α in any situations.
(3) Luteolin displayed certain extent of bi-directional regulation on IL-1β expression. But for iNOS and TNF-α, the expressions were overall reduced by luteolin. Of particular note, luteolin upregulated the expression of MCP-1 no matter with or without LPS stimulation.
(4) Salvigenin exhibited only slight inhibition against LPS-induced IL-1β expression.

Collectively, our data demonstrated the highly varied mode of immune modulation on diverse proinflammatory mediators by different active components of SBD.

## 4 Discussion

The innate immunity of host functions as a universal defense mechanism against invading pathogens nonspecifically, and the innate immune stimulators/enhancers have provided promising options to combat diverse virus infections (2). However, the protective effects of antiviral innate immunity may become detrimental when overactivated, conversely causing tissue damages, in which scenario an immune stimulator/enhancer may cause more severe disease while administration of immune suppressors instead may be beneficial (2). Of note, although an immune suppressor can alleviate the tissue damage caused by excessive innate immunity, earlier or complete immune suppression may on the other hand impair the intrinsic antiviral property. It is therefore critical to monitor the disease status of a patient to determine the appropriate timing of treatment for either innate immune enhancers or suppressors, compromising their value and translational potency (4, 5).

Previously, we reported that SBD, a Chinese herbal medicine, exhibited potential bi-directional modulatory effect *in vitro*, by promoting the transition of mouse macrophages Raw264.7 to a proinflammatory status, while suppressing the excessive inflammation induced by LPS (6). In the present study, we further validated the bi-directional immune modulatory effect of SBD *in vivo*. First, SBD can promote the proinflammatory polarization of a proportion of lung macrophages, improving the preparedness for the host to combat invading pathogens. Second, in the case of excessive inflammation, SBD on the contrary reduced the proinflammatory polarization of macrophages, alleviating tissue damages accordingly.

Actually, the host possesses an intrinsic ability to maintain the macrophage homeostasis, by finely orchestrating the expression and/or activation of diverse host factors (11). For instance, the E3 ubiquitin ligase TRIM29 serves a crucial role in the maintenance of macrophage homeostasis, by negatively regulating the expression of type-I interferons as well as pro-inflammatory mediators (12, 13). It can be supposed that these critical host factors provide promising therapeutic targets when the host immunological homeostasis is vulnerated by either internal or external pathogenic agents. It will be interesting to further elucidate the precise targets and pharmacological mechanisms underlying the bi-directional immune modulatory effect of SBD in the future.

We also investigated the material basis underlying the bi-directional modulatory effect of SBD on the inflammatory response of host, which was finely orchestrated by the combination of multi-components via potential synergistic, additive as well as antagonistic effects. In the future, it is interesting to carefully evaluate the ratio of each active compound in SBD and their contributions for the bi-directional immune modulatory property. It can be anticipated that, by mimicking the compositions of SBD, these main active components can be combined as a cocktail to obtain reasonable bi-directional immune modulatory effects, being able to ameliorate the excessive inflammation without affecting the antiviral defenses, thus harboring an expanded therapeutic window and enhanced ease-of-use clinically. Moreover, the compositions and their ratio in the cocktail can be freely adjusted to achieve a more optimized therapeutic efficacy.

In summary, our study validated the bi-directional immune modulatory property of SBD, emphasizing its value as an advanced therapeutic agent against infectious diseases. In addition, we illustrated the material basis underlying the bi-directional immune modulation of SBD, shedding light on the development of next-generation bi-directional immune modulators.

## Data Availability

The original contributions presented in the study are included in the article/Supplementary material, further inquiries can be directed to the corresponding authors.
