# Supplementary material for: In vivo evaluation and material basis of the bi-directional immune modulatory effect of Scutellaria barbata D. Don extract
Source: Front Med (Lausanne). 2026 Apr 7;13:1797041. doi: 10.3389/fmed.2026.1797041 (PMC13095528; doi:10.3389/fmed.2026.1797041)
Supplement: Supplementary file 1 [file Table_1.docx]

Supplementary Material

# Supplementary table 1. Primers used for qPCR analysis

| Primer | Sequence (5’-3’) |
| --- | --- |
| TNFα-forward | GCCTCTTCTCATTCCTGCTTG |
| TNFα-reverse | CTGATGAGAGGGAGGCCATT |
| IL6-forward | CCAGGTAGCTATGGTACTCCAGAA |
| IL6-reverse | GCTACCAAACTGGATATAATCAGGA |
| GAPDH-forward | CATTCTCGGCCTTGACTGTG |
| GAPDH-reverse | TAAATACGGACTGCAGCCCT |
| IL-1β-forward | TGTGGAGAAGCTGTGGCAG |
| IL-1β-reverse | CAGCAGGTTATCATCATCATC |
| MCP-1- forward | CTCCAGCCTACTCATTGGGATCA |
| MCP-1- reverse | GCATCCACGTGTTGGCTCA |
| iNOS- forward | TCGATGCACAACTGGGTGAAC |
| iNOS- reverse | GGAGTGACGGCAAACATGACT |

# Supplementary Figures


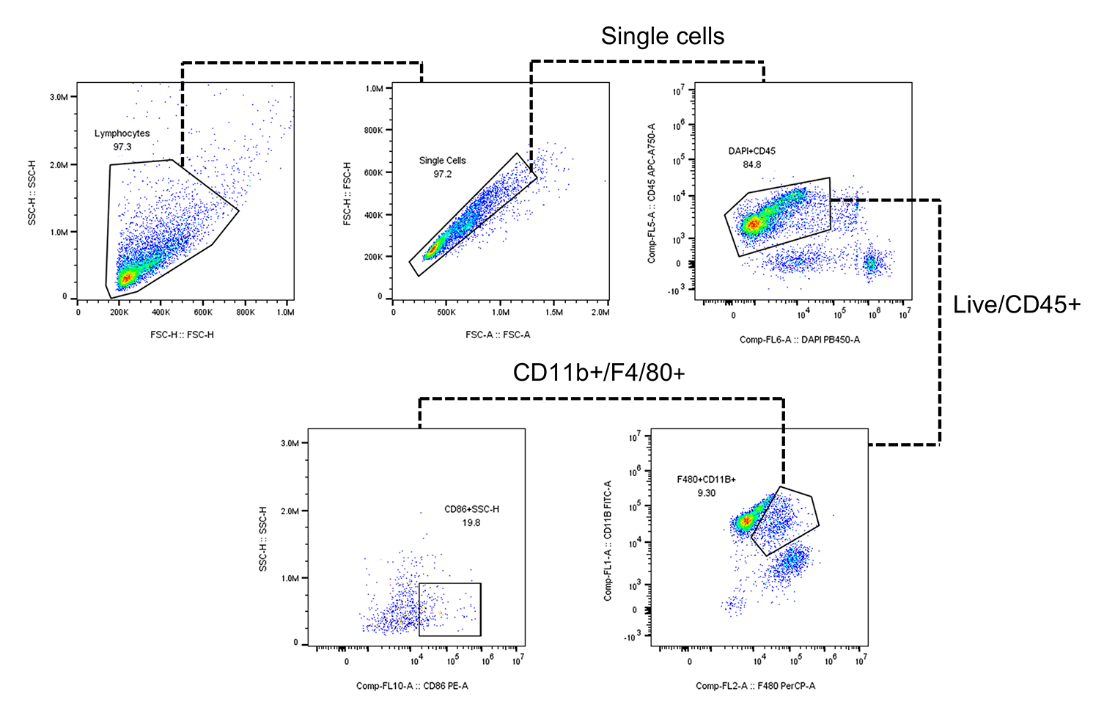


**Supplementary Figure 1.** The strategy for gating of macrophages in mice lungs and recognizing polarized inflammatory macrophages.


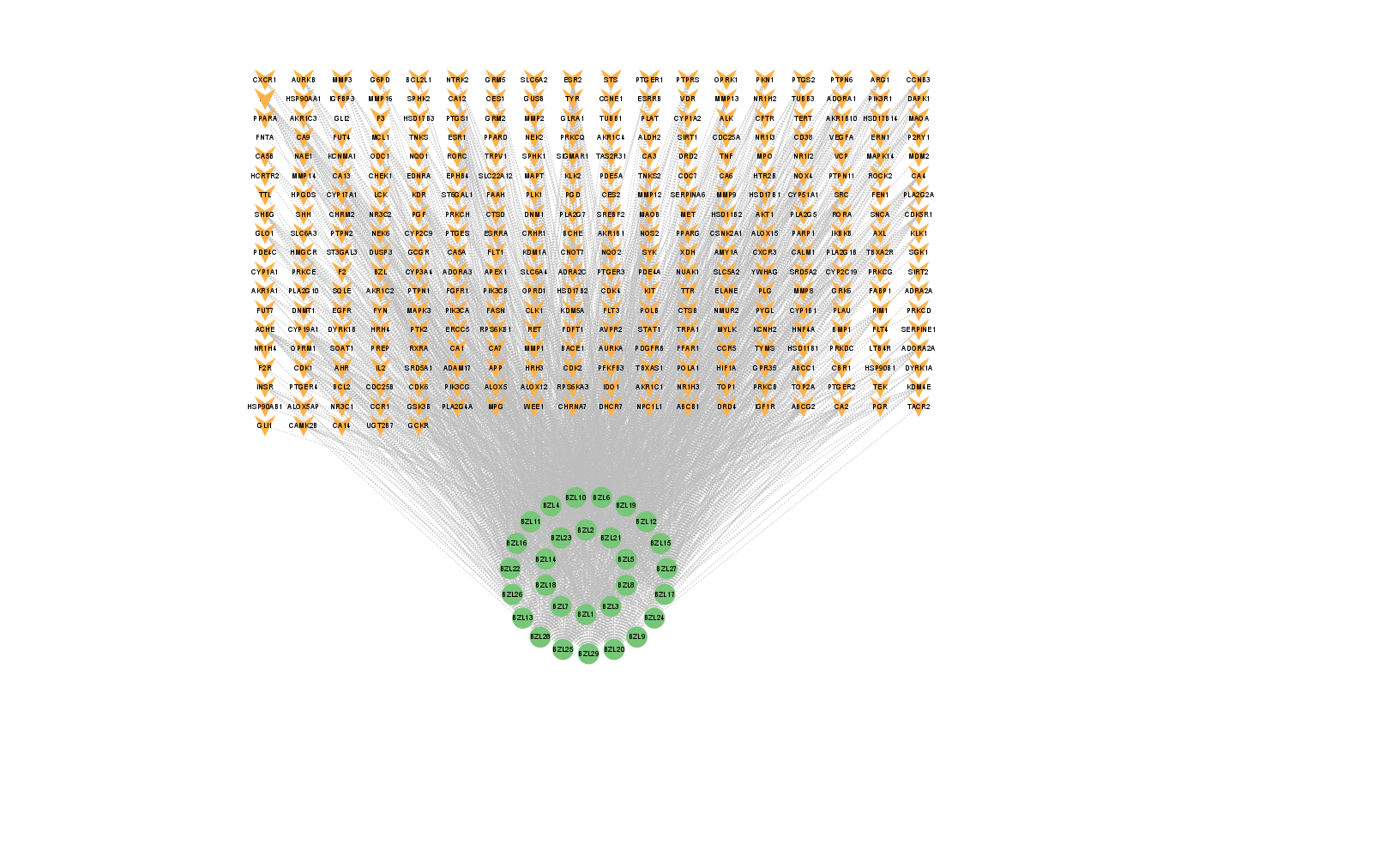


**Supplementary Figure 2.** The active ingredient-target network of SBD.


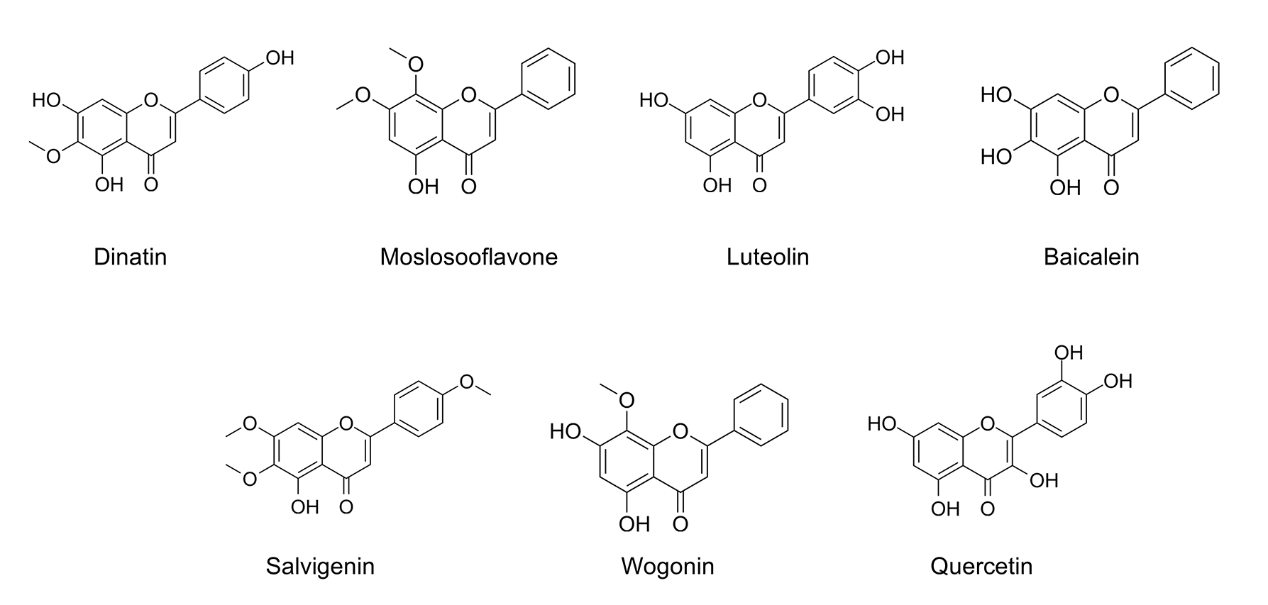


**Supplementary Figure 3.** The chemical structures of the most potential active components of SBD.
